# Research on non-destructive detection of chilled meat quality based on hyperspectral technology combined with different data processing methods

**DOI:** 10.3389/fnut.2025.1623671

**Published:** 2025-07-25

**Authors:** Zeyu Xu, Yu Han, Shuai Chen, Dianbo Zhao, Huanli Yao, Jiale Hao, Junguang Li, Ke Li, Shengjie Li, Yanhong Bai

**Affiliations:** ^1^College of Food and Bioengineering, Zhengzhou University of Light Industry, Zhengzhou, China; ^2^Key Laboratory of Cold Chain Food Processing and Safety Control, Ministry of Education, Zhengzhou University of Light Industry, Zhengzhou, China; ^3^Henan Key Laboratory of Cold Chain Food Quality and Safety Control, Zhengzhou, China; ^4^School of Food Science and Technology, Dalian Polytechnic University, Dalian, China

**Keywords:** chilled meat, hyperspectral, wavelength selection, total volatile basic nitrogen, total viable count, non-destructive detection

## Abstract

This study utilized hyperspectral technology in conjunction with chemometric methods for the non-destructive assessment of chilled meat quality. Average spectra were extracted from regions of interest within hyperspectral images and further optimized using seven preprocessing techniques: S-G, SNV, MSC, 1st DER, 2nd DER, S-G combined with SNV, and S-G combined with MSC. These optimized spectra were then incorporated into PLSR and BPNN models to predict TVB-N and TVC. The results demonstrated that the PLSR model employing S-G smoothing in combination with SNV preprocessing yielded optimal predictions for TVB-N (Correlation coefficient = 0.9631), while the integration of S-G smoothing with MSC preprocessing achieved the best prediction for TVC (Correlation coefficient = 0.9601). This methodology presents a robust technical solution for rapid, non-destructive evaluation of chilled meat quality, thereby highlighting the potential of hyperspectral technology for accurate meat quality monitoring through precise quantification of TVB-N and TVC.

## 1 Introduction

Chilled meat and its derived products represent a significant source of essential nutrients for humans. The rich composition of protein, fat, vitamins, for example, including B1, B6, etc., and other components in chilled meat closely aligns with the nutritional requirements of the human body, facilitating ease of digestion and absorption. Consequently, chilled meat possesses high nutritional value (1, 2). However, the rich nutritional matrix present in meat not only supplies energy to the human body but also acts as a natural medium conducive to the growth and reproduction of most microorganisms. This is because meat contains not only high-quality proteins, fats, amino acids, and trace minerals but also exhibits elevated water activity and content (For instance, *Pseudomonas*, Listeria, and *Lactobacillus* genera). Additionally, it maintains a natural neutral or weakly acidic pH environment, thereby providing an optimal growth setting for various microorganisms (3, 4). Moreover, chilled meat is classified as a perishable food item, which is prone to oxidation caused by microorganisms and the degradation of its own nutrients in the environment during production, distribution, or storage. This process ultimately leads to spoilage (5). TVB-N and TVC are currently the most commonly used detection indicators based on spectral non-destructive testing. Therefore, TVB-N content and TVC are critical indicators for assessing the quality of meat products. TVB-N refers to alkaline nitrogen-containing compounds, such as ammonia and amines, that are produced during the spoilage process of animal-derived food due to enzymatic and bacterial activity (6, 7). When the TVB-N content exceeds acceptable standards, it signifies that chilled meat has begun to deteriorate. Continued consumption of chilled meat at this stage may lead to a range of gastrointestinal discomforts and other adverse symptoms; in severe instances, it could even result in food poisoning (8). For example, *Salmonella* is a type of bacteria that can cause illness in humans or zoonotic diseases (known as salmonellosis). The European Union reports more than 91,000 cases of salmonellosis every year (9). TVC serves as a critical indicator for assessing the quality of chilled meat and hygiene standards. By measuring the TVC content, it is possible to ascertain whether chilled meat has been contaminated, as well as to evaluate the extent of such contamination. This assessment enables an analysis of the quality changes that occur in chilled meat during storage (10). Therefore, the quality changes of chilled meat can be effectively assessed by monitoring these two indicators. Currently, traditional methods for evaluating chilled meat quality primarily encompass sensory evaluation by humans (11), chemical index analysis (12), and microbiological assessment (13). For instance, although biosensor technology has the advantages of high sensitivity, good selectivity, rapid response and easy operation, its stability is relatively poor, it is easily disturbed by external factors, and the detection cost is also relatively high. Electromagnetic characteristic technology can achieve non-invasive detection of multiple parameters and has strong sensitivity. However, this technology is susceptible to the composition of the sample, and the equipment cost is relatively high, requiring professional operators to operate it. Computer vision technology has high detection efficiency, objectivity, and flexibility. However, at the same time, it has extremely strict requirements for technology and the environment. Its recognition ability has certain limitations, and the overall detection accuracy is average. While these methodologies boast high detection accuracy, they pose challenges for enterprises in implementing real-time, comprehensive, and rapid non-destructive testing during large-scale production (14).

To enable rapid and non-destructive quality assessment of chilled meat, researchers have conducted extensive research and proposed non-destructive testing methodologies grounded in hyperspectral technology. Hyperspectral technology, a form of spectroscopic technique, integrates the advantages of machine vision and multispectral imaging. As a result, it possesses both the high pixel imaging capabilities characteristic of standard cameras and the enhanced resolution imaging capabilities typically associated with spectrometers (15). It can continuously image the sample at 100 of wavelengths, obtain the image information and spectral information of the sample at the same time, and finally obtain the three-dimensional data block composed of two-dimensional images at different wavelengths (16). Each pixel in this 3D data block contains spectral data at different wavelengths, which includes the image information of the sample at each wavelength (17). Image information can reflect the appearance and texture features of the sample, while spectral information can reflect the physical structure and chemical composition (18).

In recent years, the combination of hyperspectral imaging technology and chemometric methods has rapidly demonstrated great potential in the field of efficient non-destructive testing of meat and its products. Hyperspectral imaging, as an innovative non-destructive testing technology, can capture both spatial information and the spectral characteristics of samples with high effectiveness (19). Hyperspectral imaging predominantly employs reflection mode, transmission mode, and diffuse reflection to acquire the three-dimensional data cube of the sample. The reflection detection method is primarily employed to analyze the external characteristics of food, encompassing parameters such as size, surface texture, and visible defects. Conversely, the transmission detection method is more appropriate for assessing the internal composition and concealed defects within the sample. Additionally, the diffuse reflectance detection method not only enables access to in-depth information about the sample but also mitigates the impact of its shape, outer surface characteristics, and thickness. Thus, it is essential to choose an appropriate mode of data collection that aligns with the specific requirements of testing and the physical characteristics of the food. This choice is critical for enhancing both the accuracy and efficiency of food quality assessments (20). At present, researchers have assessed meat quality by measuring the TVB-N content and TVC, yielding promising results. For instance, Zhang et al. (21) employed visible light near-infrared hyperspectral imaging technology to assess the total volatile nitrogen content in beef packaged with film, facilitating freshness detection. The developed model demonstrated a prediction coefficient exceeding 90% for beef across various packaging types. Liao et al. (22) developed an online detection technology utilizing visible/near-infrared (Vis/NIR) spectroscopy, which integrates the quantitative analysis of fat, protein, and moisture content in chilled meat with chemometric methods such as wavelet transform, MSC, and PLSR. This technology aims to establish an online detection system for assessing chilled meat freshness. The findings suggest that Vis/NIR spectroscopy has significant potential for the online prediction of fresh chilled meat quality. Ritthiruangdej et al. (23) used near infrared spectroscopy (NIR) technology to develop a quantitative model combining partial least squares regression (PLS) algorithm and data preprocessing methods such as MSC and second derivative to predict the main chemical composition in Thai steamed chilled meat sausage. It was found that MSC pretreatment performed well in eliminating the light scattering effect of samples, and effectively improved the prediction accuracy of the model, in which the validation set determination coefficients R^2^ of water and protein content both reached relatively ideal values. Huang et al. (24) established a BP-ANN prediction model for chilled meat TVC using 450–900 nm hyperspectral technology, and the determination coefficient *R*^2^ of the prediction set reached 0.8308. Baek et al. (25) developed a PLSR model utilizing hyperspectral images of chilled meat in conjunction with its actual TVB-N content. By integrating the feature selection methodology, they designed a shortwave infrared hyperspectral imaging system. The experimental findings indicated that the correlation coefficients *R*_c_^2^ and *R*_p_^2^ for the calibration and prediction accuracy of the optimal model were 0.94 and 0.90, respectively, demonstrating its applicability for detecting TVB-N levels in fresh chilled meat. Zhuang et al. (26) studied the quality attribute characteristics of unfrozen chilled meat using fluorescence hyperspectral imaging technology. The results showed that the prediction model based on fluorescence spectroscopy exhibited extremely high accuracy in predicting TVB-N content, with a correlation coefficient of up to 0.9447.

The primary objective of this study is to mitigate the influence of noise and environmental factors on the original spectral data by employing seven distinct preprocessing methods for spectral bands. This approach aims to enhance both the accuracy and stability of the predictive model. Secondly, the optimal feature band can be acquired through various methods of band selection. Subsequently, different modelling techniques were employed to develop a predictive model. Furthermore, there is currently no relevant literature addressing the denoising of spectral data through seven distinct preprocessing methods. Therefore, we propose the application of seven distinct spectral preprocessing methods in the development of a model for predicting chilled meat freshness. By forecasting the content indicators for TVB-N and TVC, we aim to ascertain whether the chilled meat is fresh. This establishes a theoretical foundation for the rapid, non-contact, and non-destructive assessment of chilled meat freshness.

## 2 Materials and methods

### 2.1 Sample preparation

**Figure 1:**
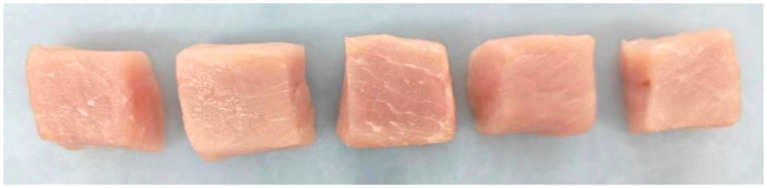
Chilled meat sample.

Purchase locally slaughtered chilled meat back 88 pieces of pork tenderloin, specifically the longest pork tenderloin, in Zhengzhou, Henan Province, China. These samples should be cooled and excreted acid for a duration of 24 h. Utilize an appropriate refrigeration device to ensure optimal conditions during transport, and promptly deliver the specimens to the laboratory within 1 h. Cut the chilled meat into pieces on a thoroughly sanitized workbench. Remove any excess fat and connective tissue, and then divide the meat into portions measuring 5 cm × 5 cm × 2 cm as illustrated in Figure 1 (The experimental instruments are presented in Table 1). Subsequently, each sample was individually stored in sterilized self-sealing bags to prevent compression and placed in a refrigerator at 4°C for a duration of 11 days. Four samples are randomly selected from the refrigerator every 12 h to gather their hyperspectral information. Once this spectral data is collected, it is immediately employed for physical and chemical value determination to ensure sample uniformity, spanning a total duration of 11 days. The sample set partitioning based on joint X-Y distances (SPXY) method is utilized to divide the samples into a calibration set and a prediction set in a 3:1 ratio (27).

**Table 1:** Instruments and equipment for chilled meat quality detection.

Instruments and equipments	Model	Manufacturer
Electronic analytical balance	XPR106DUH/AC	Mettler Toledo
Humidity chamber	SHP-250	Shanghai Hongdu Electronic Technology Co., Ltd.
Clean bench	BJ-2CD	Shanghai Boxun Industrial Co., Ltd. Medical Equipment Factory
Vertical pressure steam sterilizer	LD2M-60KCS	Shanghai Shen’an Medical Equipment Factory
High performance computational chemistry workstation	X11DAI-N	Zhengzhou 201 Technology Co., Ltd.

### 2.2 Instruments and equipment

**Figure 2:**
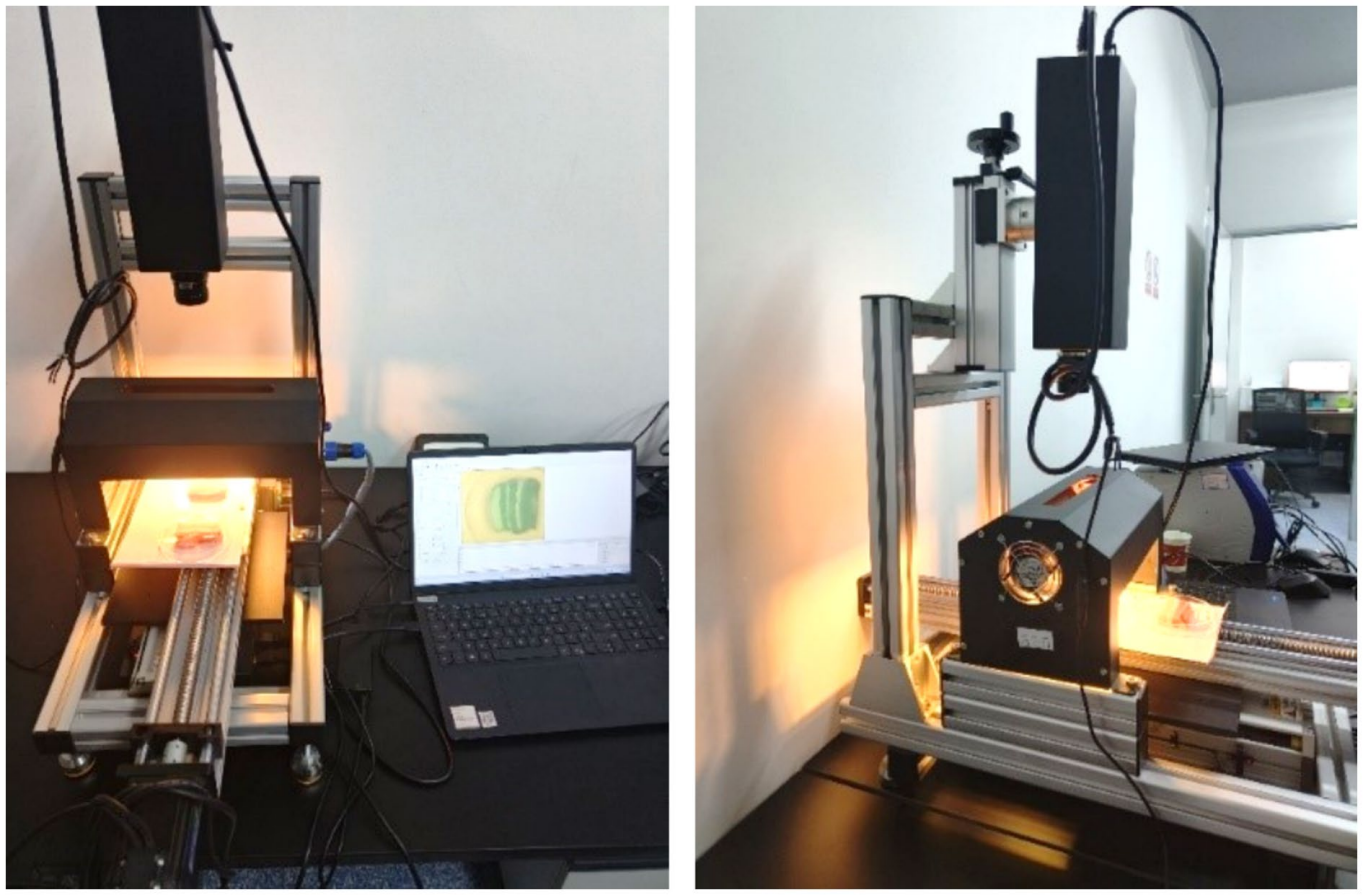
Hyperspectral imaging system.

This study employs the FigSpec series hyperspectral imaging system, which has been provided by Hangzhou Caipu Technology Co., Ltd. The system comprises a hyperspectral camera, a light source, a conveyor mechanism, spectral imaging software, and a computer system designed for data preprocessing and analysis. The acquisition device is illustrated in Figure 2.

During the experimental procedure, it is essential to first install and calibrate the light source in order to guarantee uniform and stable lighting conditions for the moving sample on the conveyor belt. Subsequently, the parameters of the hyperspectral imaging system were tuned in accordance with the experimental requirements to ensure that the acquired images encompassed the necessary spectral range and provided adequate resolution (refer to Table 2 for specific parameters of the hyperspectral camera). Initiate the conveyor belt system to ensure that the sample moves at a consistent speed and is accurately positioned within the imaging system’s field of view. When the sample traverses the imaging system, the hyperspectral camera captures its spectral image in real time. This process enables the efficient acquisition of both the spectral characteristics and spatial distribution information of the sample.

**Table 2:** Hyperspectral imaging camera parameters.

Project	Parameters
Spectral range	900–1700 nm
Spectral resolution	8 nm
Image resolution	320 × 320
Field angle	17°
Minimum exposure time	1 um
Maximum exposure time	1 s

### 2.3 Methods

#### 2.3.1 Hyperspectral image acquisition

System settings prior to the acquisition of spectral data: the spectral range was set from 900 to 1700 nm, with an exposure time of 10 ms. The exposure speed was established at 15 mm/s, and the scanning width measured 180 mm. These parameters were optimized to ensure that the resulting images are both clear and undistorted. To mitigate the effects of camera dark current, indoor lighting conditions, and detector sensitivity on the acquired images, it is essential to apply black-and-white correction in accordance with the following Equation 1 prior to sampling hyperspectral data. The acquired original hyperspectral image is calibrated to reflectance mode using both a black reference image and a white reference image.


(1) $$R=\frac{R_{0}-B}{W-B}$$

Where: R represents the value of relative reflectance following black and white correction; $R_{0}$ denotes the pixel brightness value of the original image; W indicates the pixel brightness value of the reference plate; B refers to the internal system noise present in the imaging spectrum.

#### 2.3.2 Determination of TVB-N content

According to the micro diffusion method outlined in the national food safety standard GB 5009.228-2016 for determining volatile basic nitrogen in food, TVB-N content in chilled meat was assessed. Mince the chilled meat sample thoroughly and mix it well. Accurately weigh 20 g of the sample. Transfer the weighed sample into a conical flask with a stopper. Continuously shake the conical flask while adding distilled water until reaching a total volume of 100 mL, ensuring that the sample is uniformly dispersed throughout the liquid. After allowing it to soak for 30 min, filter the resulting solution and apply a thin layer of the filtrate along the edges of the diffusion dish. Then, 1 mL of boric acid solution (boric acid obtained from Sinopharm Chemical Reagent Co., Ltd.) and 50 μL of a pre-prepared mixed indicator (comprising methylated blue and methylene blue, also sourced from Sinopharm Chemical Reagent Co., Ltd.) were introduced into the inner chamber located at the center of the dish (28). Accurately add 1 mL of the filtrate into the outer chamber and cover it with a frosted glass lid. Quickly introduce 1 mL of saturated potassium carbonate solution through the gap, and then immediately press down on the glass lid to ensure a tight seal. Then, gently rotate the diffusion dish in a circular motion to achieve thorough mixing of the sample solution with the saturated potassium carbonate solution. Place the diffusion dish in a 37°C incubator for 2 h. After incubation, allow it to cool to room temperature and then remove the cover. Titrate the dish with a hydrochloric acid standard titration solution (hydrochloric acid is procured from Sinopharm Chemical Reagent Co., Ltd.). The indicator comprises a mixed solution consisting of two parts methyl red ethanol solution and one part methylene blue ethanol solution. The color observed at the endpoint of the titration is bluish purple. At the same time, a reagent blank control was conducted. The calculation method is illustrated in Equation 2:


(2) $$X=\frac{(V_{1}-V_{2})\times c\times 14}{m}\times 100$$

Where: *X* represents the TVB-N content in the sample, expressed in mg/100 g; *V*_1_ denotes the volume of hydrochloric acid utilized by the measured sample, measured in mL; *V*_2_ indicates the volume of hydrochloric acid used by the blank sample, also measured in mL; *c* signifies the concentration of hydrochloric acid standard titration solution, provided in mol/L; *m* refers to the mass of the measured sample, indicated in g.

Evaluation standard: In accordance with the national food safety standard GB 2707-2016, fresh meat is defined as having a volatile basic nitrogen content of less than or equal to 15 mg/100 g.

#### 2.3.3 Determination of TVC content

The colonies were cultured and enumerated in accordance with the national food safety standard GB 4789.2-2022, which outlines the “determination of the total number of colonies in the microbiological examination of food.” A sample weighing 25 g was placed into a sterile homogenization bag containing 225 mL of phosphate buffer. The mixture was then subjected to agitation using a flapping homogenizer for 2 min to prepare a 1:10 sample homogenate. Use a 1 mL micropipette to withdraw 1 mL of the 1:10 sample homogenate. Carefully inject this into a sterile test tube containing 9 mL of diluent along the wall of the tube. Gently shake the test tube to ensure thorough mixing, thereby preparing a 1:100 sample homogenate. Following this procedure, prepare subsequent dilutions of 1:1,000, 1:10,000, and 1:100,000 in sequence. When performing tenfold incremental dilution, aspirate 1 mL of the sample homogenate into a sterile plate and prepare two plates for each dilution. At the same time, add 1 mL of the blank diluent into two sterile plates to serve as blank controls. Pour 15–20 mL of agar plate counting medium, cooled to 46°C, into a Petri dish (sourced from China National Pharmaceutical Group Chemical Reagent Co., Ltd.), and gently rotate the Petri dish to ensure even distribution. After allowing the agar to solidify, invert the plate and incubate it at 36°C for a period of 48 h. The total number of bacterial colonies is calculated according to Equation 3:


(3) $$N=\frac{\sum C}{(n_{1}-0.1n_{2})d}$$

In the formula, *N* represents the total number of bacterial colonies present in the sample; ∑*C* denotes the sum of suitable plate bacterial counts; *n*_1_ indicates the colony count from the first dilution plate; *n*_2_ refers to the colony count from the second dilution plate; and *d* signifies the dilution factor.

The experimental results are expressed as the logarithm of the total bacterial count. The evaluation criteria are established according to the domestic trade industry standard SB/T 10482-2008 of the People’s Republic of China, which stipulates that the total bacterial count for fresh meat should not exceed 1 × 10^6^ CFU/g.

### 2.4 Spectral data processing and analysis

#### 2.4.1 Spectral data preprocessing

**Figure 3:**
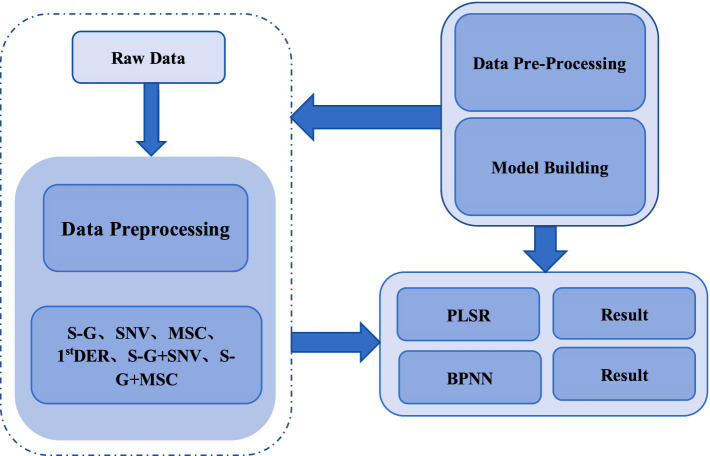
Spectral data preprocessing methods and later model construction framework.

In this study, the hyperspectral image data were processed using ENVI 5.3 software. Firstly, open the hyperspectral image data of the calibrated chilled meat sample. Employ a rectangular selection tool to define a 200 × 200 pixel area near the center of each sample as the Region of Interest (ROI). Subsequently, extract the average spectral data from this ROI to obtain the original spectral information for the sample (29). To ensure the accuracy and reliability of hyperspectral data, the collected hyperspectral images must undergo a series of multi-step preprocessing operations once they are transmitted to the computer system. Firstly, dark frame correction is conducted by subtracting the dark frame image to eliminate system noise and artifacts. Subsequently, flat field correction is executed by dividing the hyperspectral image by the flat field image, thereby compensating for the non-uniformity of both the sensor and the light source. Spectral calibration is employed to guarantee the precise allocation of wavelengths for each pixel, while geometric correction serves to spatially align the image, thereby eliminating potential distortions. However, in addition to providing valuable information regarding the sample, hyperspectral raw data also includes a significant amount of redundant information, such as light scattering and collinearity data. It is essential to conduct spectral preprocessing on the raw data in order to enhance the robustness and accuracy of the predictive model. This study used seven spectral preprocessing methods, including Savitzky-Golay (S-G) smoothing, Standard Normal Variate Transformation (SNV), Multiplicative Scatter Correction (MSC), First Derivatives (1st DER), Second Derivatives (2nd DER), S-G combined with SNV, and S-G combined with MSC, to optimize data quality and improve model prediction performance. As shown in Figure 3, the spectral data preprocessing method, and the framework for constructing the later model are presented.

#### 2.4.2 Model building

**Figure 4:**
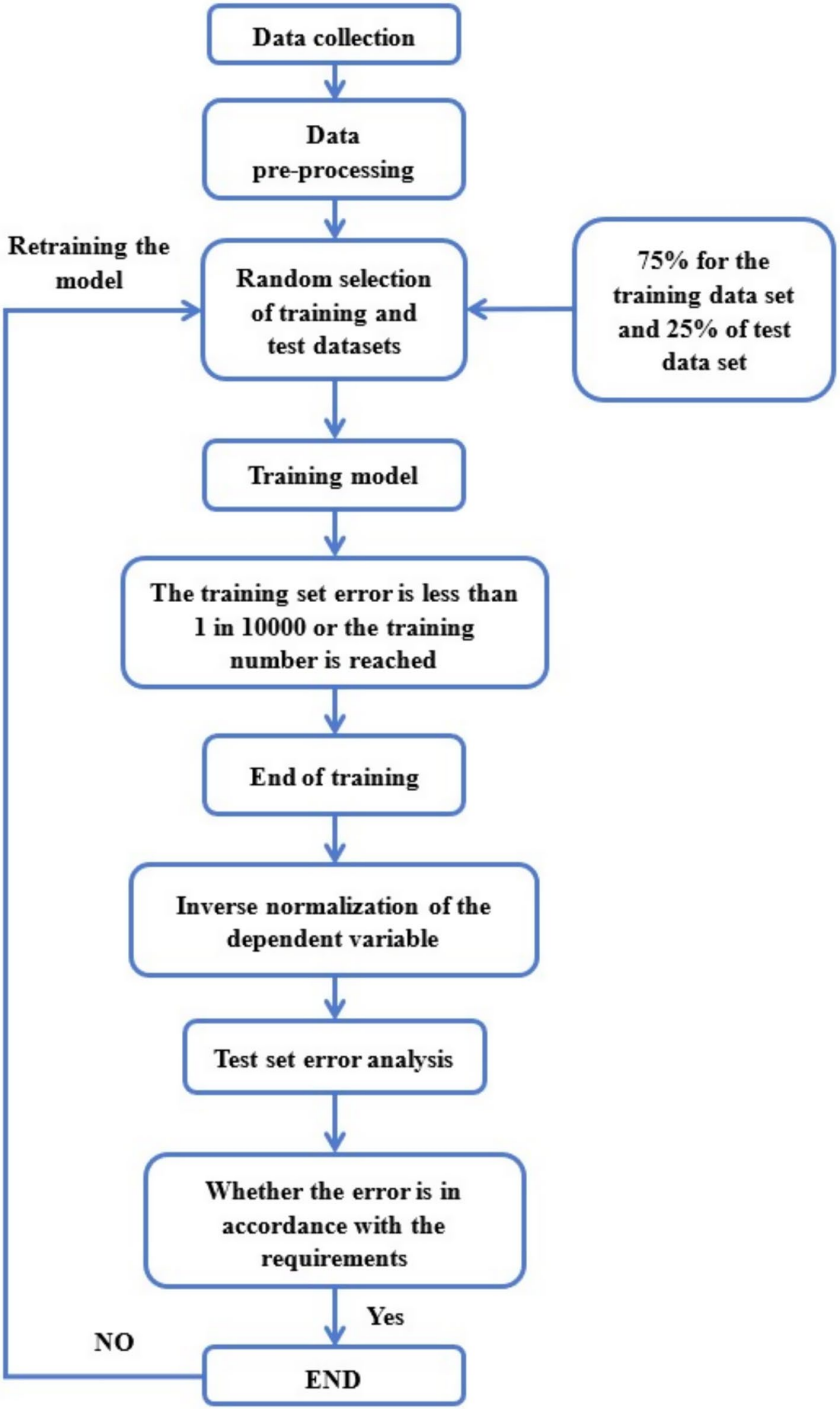
Logic process of the BP neural network algorithm.

**Figure 5:**
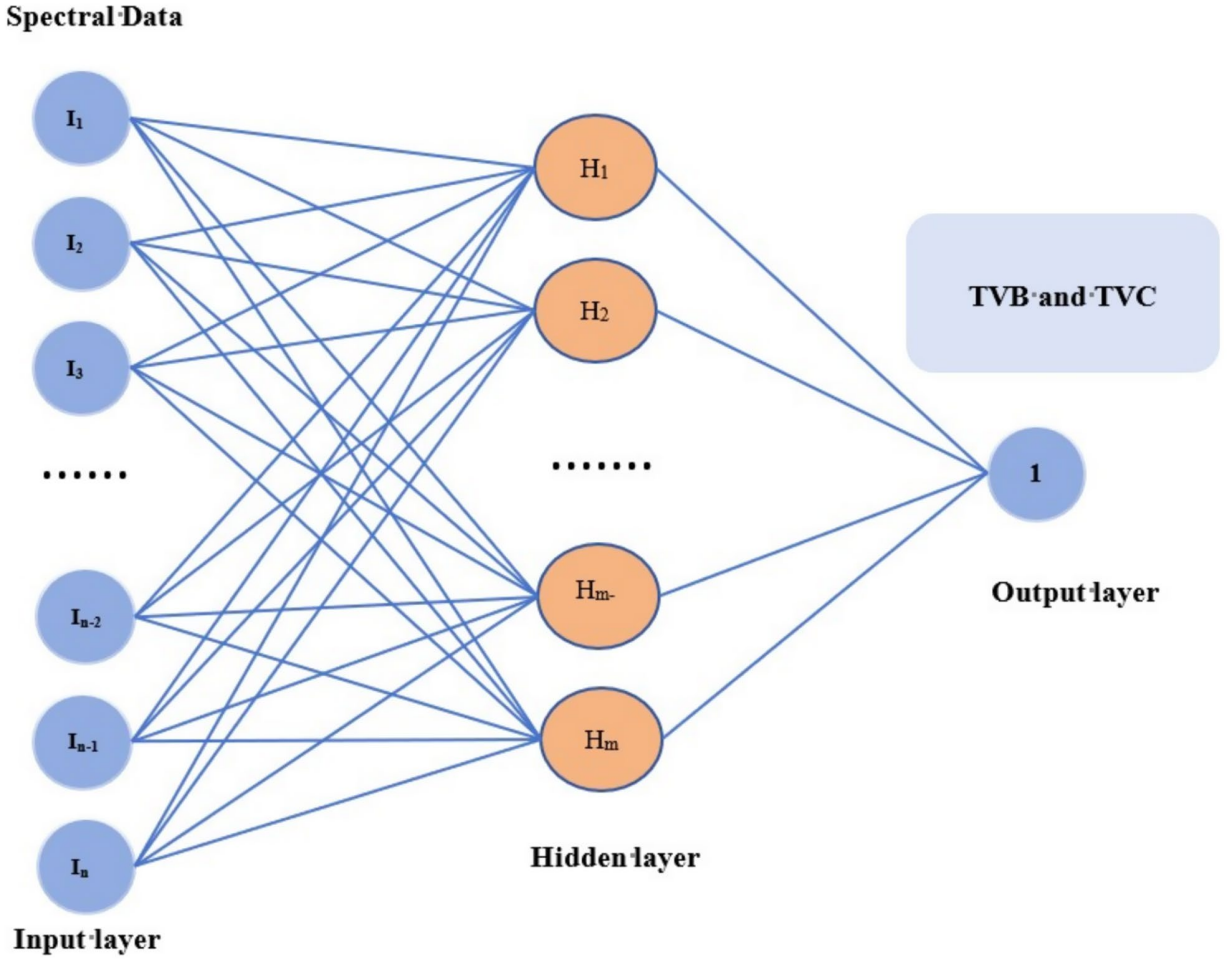
Establishment of the BP neural network model.

The spectral data contains information on the structure and composition of chilled meat, which is related to its quality parameters. In this study, PLSR and BP neural network algorithms were used to construct the model. As shown in Figures 4, 5, where the Logic process of the BP neural network algorithm in the figure dataset and the establishment of the BP neural network model in the figure. The Hyperspectral Information of samples and their quality parameters can be correlated by establishing hyperspectral technology and chemometrics model to determine the quantitative relationship between them.

## 3 The results and analysis

### 3.1 Hyperspectral data analysis

**Figure 6:**
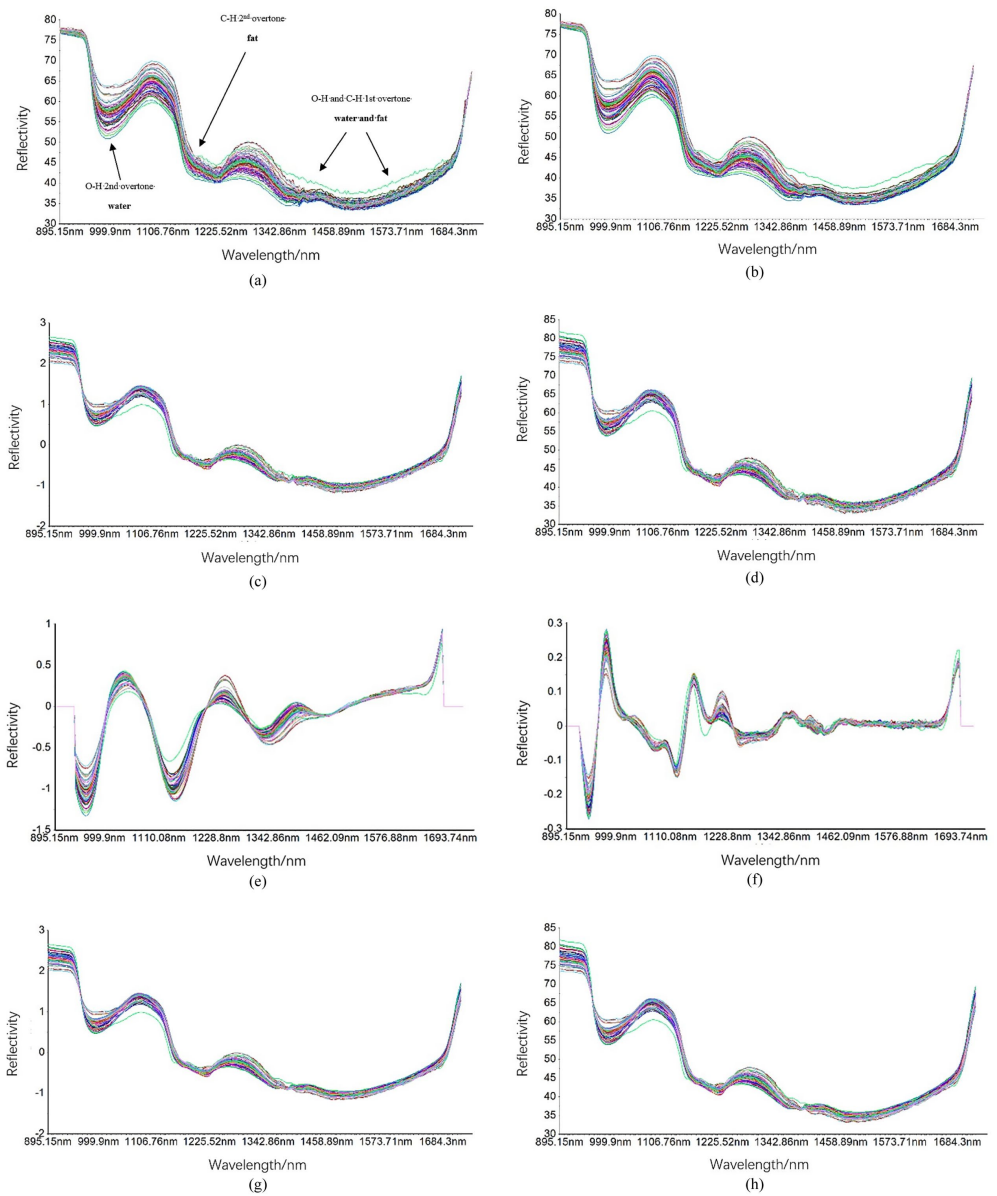
Spectral curves preprocessed using different methods. Including: **(a)** original spectral curve, **(b)** S-G preprocessed spectral curve, **(c)** SNV preprocessed spectral curve, **(d)** MSC preprocessed spectral curve, **(e)** 1st DER preprocessed spectral curve, **(f)** 2nd DER preprocessed spectral curve, **(g)** S-G + SNV preprocessed spectral curve, and **(h)** S-G + MSC preprocessed spectral curve.

Utilize ENVI5.3 software to acquire the average spectral data for the region of interest from the collected hyperspectral images. The original spectral data of the sample were processed utilizing techniques such as S-G smoothing, SNV, MSC, 1st DER, 2nd DER, S-G combined with SNV, and S-G combined with MSC. This processing yielded the spectral curves illustrated in Figure 6.

According to the analysis presented in Figure 6, the spectral data following S-G smoothing preprocessing exhibit peaks at wavelengths of 1,000 nm, 1,106 nm, and 1,285 nm. In contrast, the spectral data processed using SNV show peaks at wavelengths of 1,006 nm, 1,100 nm, and 1,287 nm. The spectral data subjected to MSC reveal peaks at wavelengths of 989 nm, 1,088 nm, and 1,282 nm. Furthermore, the first derivative extracted from the spectral data after 1st DER displays distinct peaks at wavelengths of 950 nm, 1,046 nm, 1,151 nm, 1,254 nm, 1,339 nm, and 1,435 nm. Meanwhile, the second derivative obtained through 2nd DER shows notable peaks at wavelengths of 951 nm, 989 nm, 1,129 nm, 1,158 nm, 1,202 nm, 1,217 nm, and 1,374 nm. Overall analysis indicates a prominent peak exists at these specific wavelength ranges. The combination of S-G with SNV and the S-G combined with MSC demonstrates a superior capacity to mitigate noise. The preprocessed spectral data for S-G combined with SNV exhibits distinct peaks at wavelengths of 1,009 nm, 1,090 nm, and 1,290 nm, whereas the preprocessed spectral data for S-G combined with MSC reveals prominent peaks at wavelengths of 991 nm, 1,090 nm, and 1,287 nm.

### 3.2 Based on the analysis of conventional detection methods chilled meat quality indicators

#### 3.2.1 Analysis of TVB-N content change

**Figure 7:**
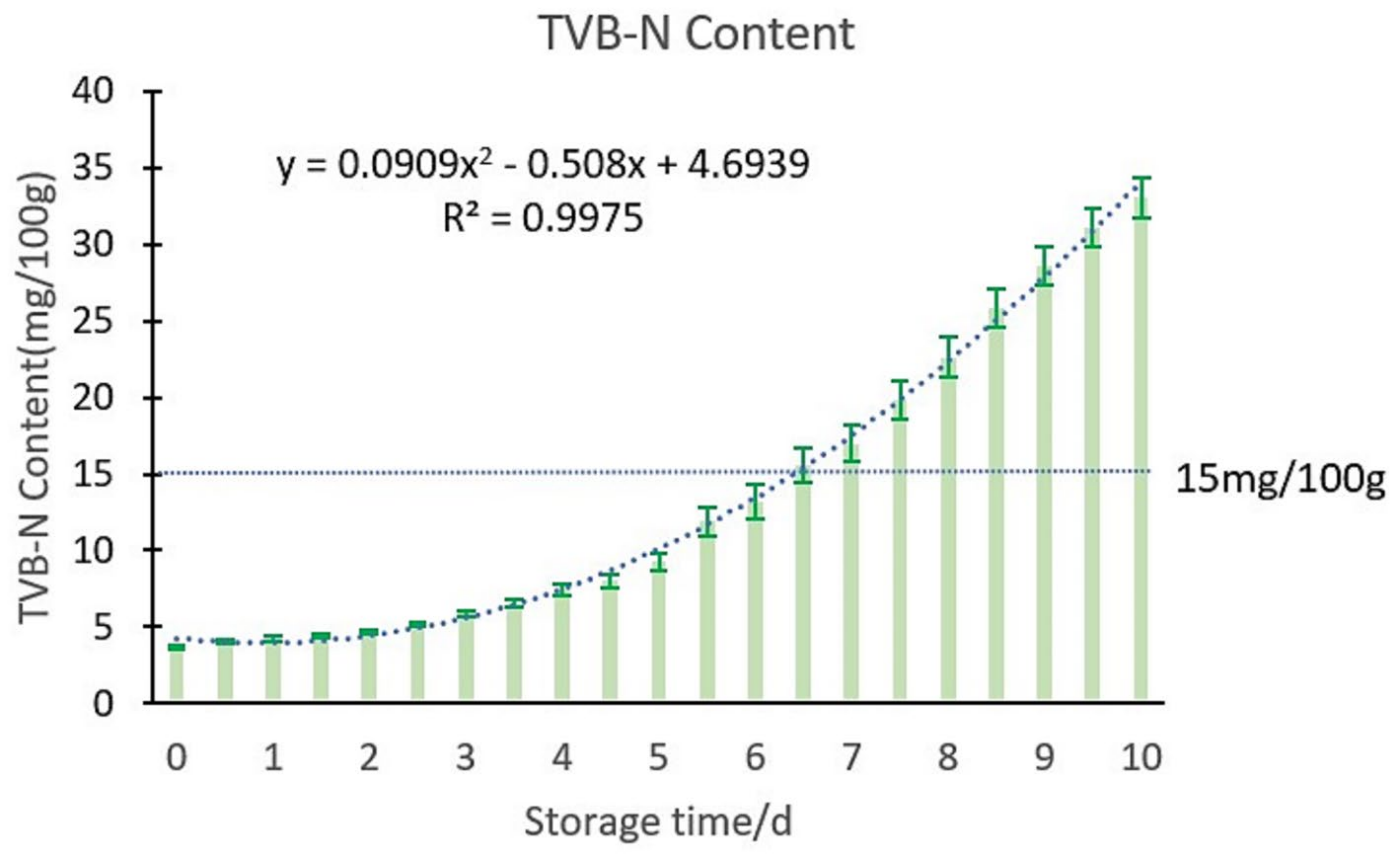
Changes of TVB-N content in chilled meat during storage.

According to the section 2.3.2 the steps of the preparation of test samples, according to the method of GB 5009.228 2016 to test the chilled meat TVB-N content. As a high-protein food, livestock and poultry meat undergoes decomposition into volatile amine compounds, such as trimethylamine, dimethylamine, and ammonia, during the spoilage process. Collectively, these substances are referred to as TVB-N. The content of TVB-N serves as a crucial indicator for assessing the extent of spoilage in high-protein foods and evaluating the freshness of meat products. When the content of TVB-N exceeded 15 mg/100 g, fresh meat spoilage. In this study, chilled meat samples were stored at 4°C for 11 days, during which the dynamic changes in TVB-N content were monitored. The results are shown in Figure 7.

According to Figure 7, the TVB-N content in chilled meat exhibits an increasing trend with extended storage duration. This upward trajectory can be attributed to the growth and reproduction of various microorganisms, which result in protein degradation and the formation of nitrogenous compounds, such as ammonia and amines. Consequently, these processes lead to a gradual increase in TVB-N levels. In this study, the TVB-N content in fresh chilled meat was found to be approximately 4 mg/100 g. When the TVB-N level exceeds 15 mg/100 g on the sixth and a half day under refrigeration conditions at 4°C, it indicates that the chilled meat has transitioned from being classified as fresh meat to either sub-fresh or spoiled meat.

#### 3.2.2 Analysis of TVC changes

**Figure 8:**
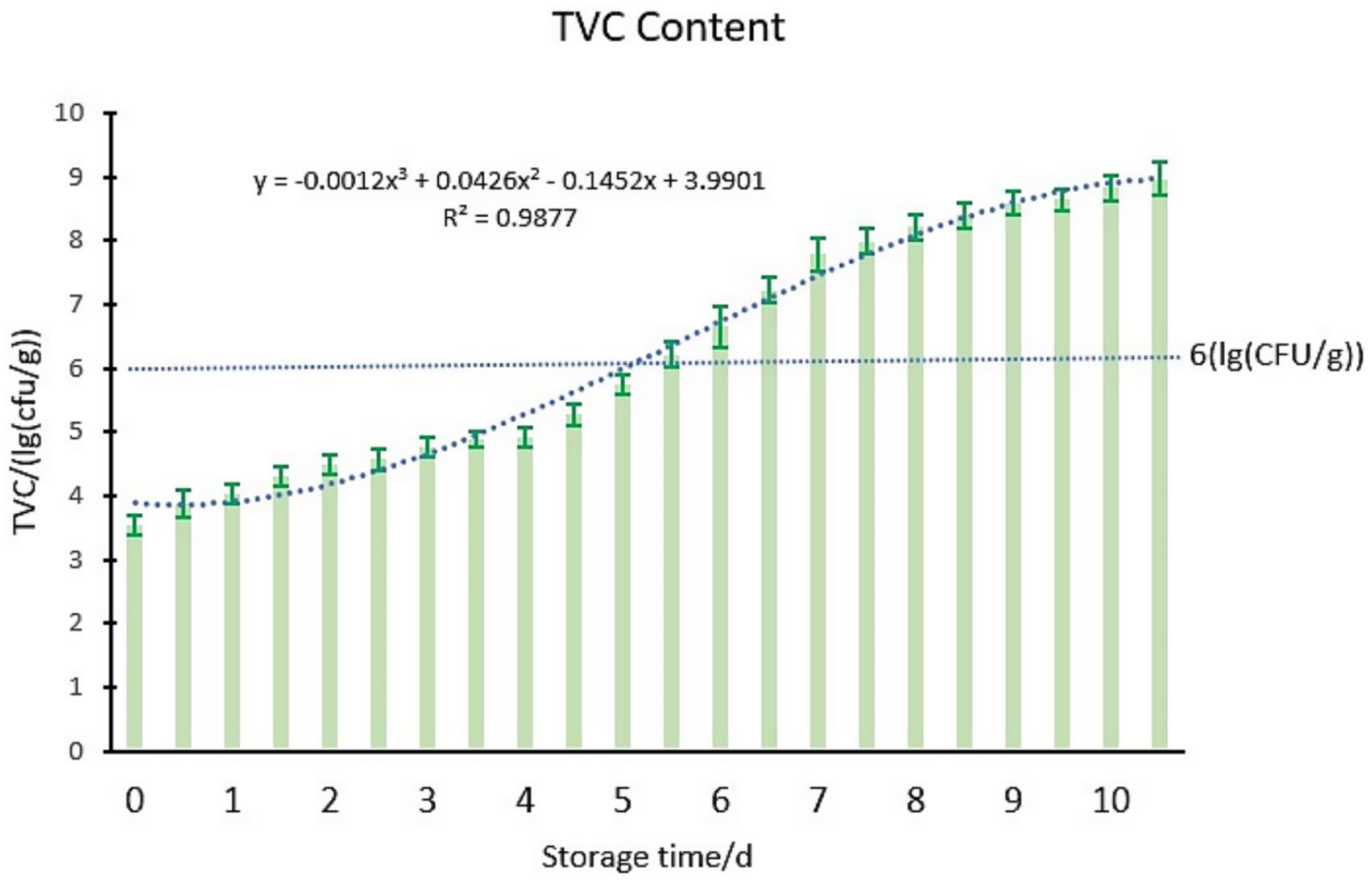
Changes of TVC in chilled meat during storage.

According to the section 2.3.3 the steps of the preparation of test samples, according to the method of GB 4789.2-2016 to test the chilled meat TVC content. Microorganisms serve as a critical indicator for assessing the freshness and safety of fresh meat. They can induce alterations in both the appearance and safety of food products. According to the standard outlined in SB/T 10482-2008, the meat begins to spoil and deteriorate when the total bacterial count exceeds 6.00 lg (CFU/g). The chilled meat samples were stored at 4°C for a duration of 11 days, with the corresponding changes in TVC content throughout the storage period illustrated in Figure 8.

As illustrated in Figure 8, the increase in storage time leads to a significant proliferation of microorganisms in chilled meat, resulting in a corresponding upward trend in TVC. The initial TVC value is recorded at 3.53 lg (CFU/g), indicating that it remains within the lag phase. By the fourth day, the count transitions into the logarithmic phase, and by the fifth day, it surpasses 6.00 lg (CFU/g), signifying evident spoilage and decay of meat samples under investigation.

### 3.3 Based on the analysis of high spectrum detection method of chilled meat quality indicators

#### 3.3.1 TVB-N content modelling and analysis

After preprocessing the original spectral data in MATLAB R2022b software, Construct PLSR and BPNN models for the spectral data obtained from each preprocessing method, separately. After regression analysis, the TVB-N index regression model was obtained. To verify the quality index prediction model, the correlation coefficient (*R*), root mean square error of cross validation (RMSECV), and root mean square error of prediction (RMSEP) of the chilled meat TVB-N index regression model were calculated. The evaluation parameter results of the model are shown in Table 3.

**Table 3:** Model evaluation parameters of chilled meat’s TVB-N.

Modelling method	Preprocessing method	Correlation coefficient	Root mean square error of cross validation	Root mean square error of prediction
		*R*	RMSECV	RMSEP
PLSR	Unpreprocessed	0.8912	0.9152	1.1218
	S-G	0.9236	0.7832	1.0211
	SNV	0.9386	0.5099	0.9127
	MSC	0.9323	0.5128	0.9836
	1st DER	0.9212	0.6589	1.0255
	2nd DER	0.9258	0.6326	1.1244
	S-G + SNV	0.9631	0.4427	0.7433
	S-G + MSC	0.9574	0.4631	0.7765
BPNN	Unpreprocessed	0.8804	0.9217	1.8221
	S-G	0.9034	0.8521	1.6215
	SNV	0.9117	0.8124	1.5243
	MSC	0.9203	0.7854	1.4835
	1st DER	0.9027	0.8835	1.6354
	2nd DER	0.8976	0.8921	1.7146
	S-G + SNV	0.9413	0.6134	1.3476
	S-G + MSC	0.9311	0.6785	1.3755

**Figure 9:**
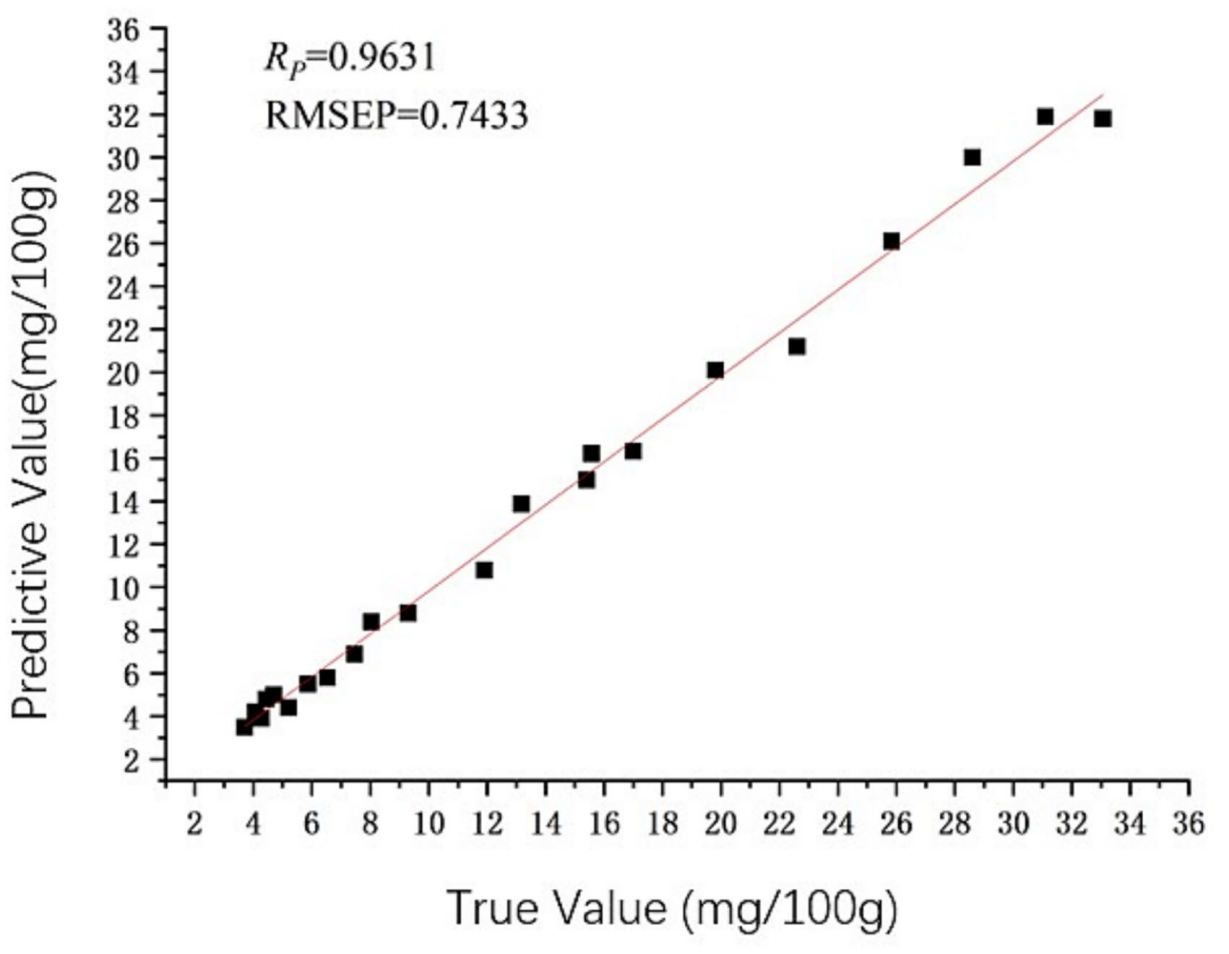
Predicted vs. measured values of TVB-N Using PLSR (S-G + SNV).

According to Table 3, the evaluation parameters of the spectral data wavelength without any preprocessing were compared with those obtained from the PLSR and BPNN models developed through seven distinct preprocessing methods applied to the spectral wavelength. The evaluation metrics for the model constructed using untreated spectral data were found to be inferior when compared to those derived from the models utilizing any of the seven specified preprocessing techniques. Clearly, the seven preprocessing methods are advantageous in enhancing the predictive performance of the model. Meanwhile, the chilled meat TVB-N index model, which was constructed utilizing the S-G method combined with SNV preprocessing, exhibited superior performance. Overall, the predictive capability of the PLSR model slightly outperformed that of the BPNN model. The optimal model for the chilled meat TVB-N index is identified as the PLSR model, which utilizes S-G smoothing in conjunction with SNV pretreatment. This model demonstrates a *R* of 0.9631, a RMSECV of 0.4427, and a RMSEP of 0.7433. Figure 9 illustrates the scatter plot comparing the measured values with those predicted by the best-performing model for TVB-N content.

#### 3.3.2 Modelling and analysis of TVC

After preprocessing the original spectral data in MATLAB R2022b software, Construct PLSR and BPNN models for the spectral data obtained from each preprocessing method, separately. After regression analysis, the TVC index regression model was obtained. To verify the quality index prediction model, the correlation coefficient (*R*), root mean square error of cross validation (RMSECV), and root mean square error of prediction (RMSEP) of the chilled meat TVC index regression model were calculated. The evaluation parameter results of the model are shown in Table 4.

**Table 4:** Model evaluation parameters of chilled meat’s TVC.

Modelling method	Preprocessing method	Correlation coefficient	Root mean square error of cross validation	Root mean square error of prediction
		*R*	RMSECV	RMSEP
PLSR	Unpreprocessed	0.9103	0.8943	1.4285
	S-G	0.9324	0.7854	1.3024
	SNV	0.9317	0.7201	1.2865
	MSC	0.9472	0.6923	1.1747
	1st DER	0.9401	0.7138	1.2096
	2nd DER	0.8444	0.9147	1.3767
	S-G + SNV	0.9575	0.5877	0.9946
	S-G + MSC	0.9601	0.5534	0.9122
BPNN	Unpreprocessed	0.8812	1.1764	1.6788
	S-G	0.9114	0.8847	1.5753
	SNV	0.9247	0.7698	1.5012
	MSC	0.9301	0.7135	1.4428
	1st DER	0.9003	0.8124	1.6133
	2nd DER	0.8998	1.0278	1.6452
	S-G + SNV	0.9331	0.7022	1.4992
	S-G + MSC	0.9411	0.6884	1.4786

**Figure 10:**
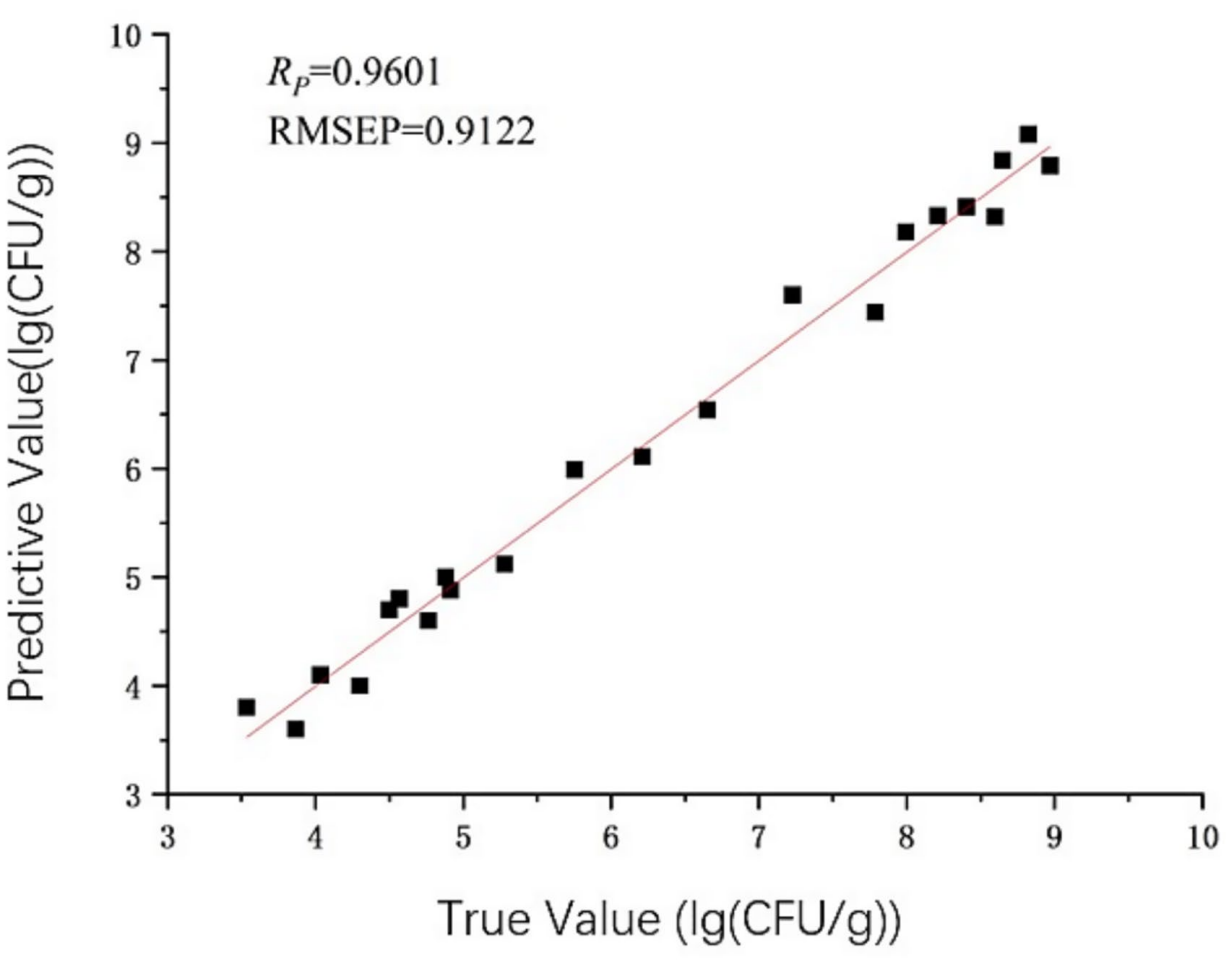
Predicted vs. measured values of TVC Using PLSR Model (S-G + MSC).

According to Table 4, the evaluation parameters of spectral data wavelengths that underwent no preprocessing were compared with those of the PLSR and BPNN models developed through seven distinct preprocessing methods applied to the spectral wavelengths. The evaluation metrics for the model constructed using untreated spectral data were found to be inferior to those of the models generated through the seven preprocessing techniques. Evidently, the seven pretreatment methods previously mentioned markedly improve the predictive performance of the model. Meanwhile, the chilled meat TVC index model constructed utilizing the S-G method in conjunction with MSC preprocessing demonstrated superior performance. Overall, the predictive performance of the PLSR model demonstrates a slight superiority over that of the BPNN model. The optimal model for predicting the TVC index of chilled meat is identified as the PLSR model following S-G method in conjunction with MSC preprocessing, which yields a correlation coefficient *R* of 0.9601, a RMSECV of 0.5534, and a RMSEP of 0.9122. The superior performance of the S-G and MSC combination may result from their complementary effects: S-G smoothing reduces noise while preserving key spectral features, and MSC corrects baseline and scatter variations. This synergy helps extract microbe-related spectral information more effectively, enhancing model robustness and generalizability. Figure 10 shows the scatter plot of the measured values of TVC content and the best model predicted values.

### 3.4 Comparative analysis of models

In this study, the PLSR model demonstrated a marginally superior performance compared to the BPNN model in predicting TVB-N and TVC. This discrepancy can be ascribed to a variety of factors. Firstly, BPNN models typically exhibit greater complexity, particularly when applied to small datasets. This heightened complexity consequently raises the likelihood of overfitting (30). In instances where the sample size is limited, BPNN models may unintentionally capture noise rather than authentic patterns, consequently diminishing their ability to generalize effectively (31). On the other hand, PLSR as a linear regression technique, achieves dimensionality reduction by extracting principal components while emphasizing significant features (32). This approach effectively reduces the risk of overfitting and enhances the stability of predictions in scenarios characterized by smaller sample sizes. Consequently, it demonstrates superior performance compared to BPNN in TVB-N and TVC prediction tasks.

In addition, spectral data frequently displays prominent linear characteristics, thereby conferring an advantage to PLSR in the analysis of hyperspectral information (33). By applying linear transformation to extract principal components and establishing a regression model based on these components, PLSR fits well with the inherent linear relationship in the spectral dataset (34). When predicting TVB-N and TVC values, PLSR effectively identified significant trends in the variations of spectral data and established robust correlations between these variations and quality indicators such as TVB-N and TVC. This approach has consequently enhanced the accuracy of predictions. Conversely, BPNN’s dependence on complex nonlinear maps may hinder its ability to adapt to the underlying linear structure of the spectral data, thus negatively impacting its prediction accuracy. Although the BPNN did not exhibit significant performance in this study, it possesses considerable potential for adapting to the nonlinear characteristics of large spectral datasets (35).

At the same time, the RER of the TVB-N model is 20.2 and the RPD is 3.8. According to the criteria in the relevant literature of meat prediction models (36), RER > 15.0 indicates that the model has excellent prediction ability, and RPD ≥ 3.0 represents that the model has high robustness and generalization ability. This result further verifies the reliability of the TVB-N model in the industrial rapid detection scenario. The RER of the TVC model was 16.5 (>15.0), but the RPD was 2.2, which was within the range (2.0 ≤ RPD ≤ 3.0), indicating that the model has a very good predictive ability and is suitable for precise quantitative analysis. Future research can further optimize the accuracy of the TVC prediction model by introducing time series features or nonlinear algorithms.

## 4 Conclusion

This study first collected hyperspectral data of chilled meat samples, and then used conventional detection methods to detect the TVB-N content and TVC produced by the same chilled meat samples at different storage times. At the same time, seven preprocessing methods were used to process the preprocessed spectral data. The preprocessed spectral data was combined with actual indicator detection values for analysis, and a TVB-N content and TVC quality prediction model based on PLSR and BPNN was constructed. The final research results indicate that:

1. Under the same preprocessing type conditions, the predictive performance of the PLSR model is slightly better than that of the BPNN model; Under the condition of the same model type, compared with the model evaluation parameters without pretreatment, several pretreatment methods have improved the prediction effect of the model. Among the PLSR model types, the chilled meat TVB-N content model predicted by the S-G combined with SNV preprocessing method had the best performance, with a correlation coefficient *R* of 0.9631, RMSECV of 0.4427, and RMSEP of 0.7433; The chilled meat TVC model with S-G combined with MSC preprocessing method has the best predictive performance, with a correlation coefficient *R* of 0.9601, RMSECV of 0.5534, and RMSEP of 0.9122.
2. Under storage conditions of 4°C, the TVB-N content of chilled meat exceeded 15 mg/100 g on the 6.5th day, indicating that the chilled meat had changed from fresh meat to less fresh or spoiled meat.
3. Under storage conditions of 4°C, the TVC of chilled meat exceeded 6.00 lg (CFU/g) on day 5.5, resulting in spoilage of test meat samples.
4. From this, the content of TVC will exceed the standard value earlier than TVB-N, and both can well reflect the quality changes of chilled meat under different storage times.

The detection method in this study has the advantages of short detection time and high efficiency compared to conventional detection methods, indicating that hyperspectral technology can be used for rapid non-destructive testing of chilled meat quality indicators. This study can provide theoretical basis and technical support for the better application of hyperspectral technology in non-destructive testing of chilled meat quality.

## Data Availability

The original contributions presented in the study are included in the article/supplementary material, further inquiries can be directed to the corresponding author.
